# mGluR_1,5_ activation improves network asynchrony and GABAergic synapse attenuation in the amygdala: implication for anxiety-like behavior in DBA/2 mice

**DOI:** 10.1186/1756-6606-5-20

**Published:** 2012-06-09

**Authors:** Fengyu Zhang, Bei Liu, Zhuofan Lei, Jin-Hui Wang

**Affiliations:** 1 State Key Laboratory, Institute of Biophysics, Chinese Academy of Sciences, 15 Datun Road, Beijing, 100101, China; 2 College of Life Science, University of Science and Technology in China, Hefei, Anhui, 230026, China

**Keywords:** Anxiety, Amygdala, GABA, Neuron, Synapse and neural network

## Abstract

Anxiety is a prevalent psychological disorder, in which the atypical expression of certain genes and the abnormality of amygdala are involved. Intermediate processes between genetic defects and anxiety, pathophysiological characteristics of neural network, remain unclear. Using behavioral task, two-photon cellular imaging and electrophysiology, we studied the characteristics of neural networks in basolateral amygdala and the influences of metabotropic glutamate receptor (mGluR) on their dynamics in DBA/2 mice showing anxiety-related genetic defects. Amygdala neurons in DBA/2 high anxiety mice express asynchronous activity and diverse excitability, and their GABAergic synapses demonstrate weak transmission, compared to those in low anxiety FVB/N mice. mGluR_1,5_ activation improves the anxiety-like behaviors of DBA/2 mice, synchronizes the activity of amygdala neurons and strengthens the transmission of GABAergic synapses. The activity asynchrony of amygdala neurons and the weakness of GABA synaptic transmission are associated with anxiety-like behavior.

## Introduction

Anxiety, characterized as unstable mood, elevated attention, negative interpretation and social phobia under the conditions of potential threatening signs, is one of prevalent psychological disorders [1-3]. The studies by neural imaging indicate the hyperactivity of amygdala in anxiety disorder [4,5]. A stimulus to amygdala induces anxiogenic somatic and autonomic responses [6]. The grafts of GABAergic-rich neural tissue into amygdala improve anxiety-like signs [7]. The abnormality of amygdala is presumably a major origin of anxiety pathogenesis [1,4,8-24]. However, anxiety-related pathological characteristics and mechanisms in amygdala remain to be elusive.

In terms of molecular mechanisms, the defects of certain genes are presumably associated with anxiety disorder [25-34]. At the cellular level, the abnormalities of amygdala neurons [1,15,22,35-37] and GABAergic synapses [38-40] are likely related to anxiety. It remains unclear how these genetic deficits lead to the impairment of amygdala neural microcircuits in anxiety disorders. In DBA/2 mice that are anxiety-like phenotype and gene variances in amygdala [29], we investigated the pathophysiological characteristics and pharmacological improvement of neuronal networks and GABAergic synapses in basolateral amygdala by two-photon cellular imaging, electrophysiology and pharmacology.

## Results

In order to study how genetic deficits impair neuronal networks in amygdala and lead to anxiety disorder, we have to select an appropriate model of animals that show anxiety-related phenotype and genotype. Compared to FVB/N mice, DBA/2 mice show anxiety-like behavior and anxiety-associated genetic variance, such as the abnormal expressions of glyoxalase-1 and glutathione reductase-1 genes in amygdala [29]. These two strains of mice were used to study the correlation among anxiety, genes and amygdala neuron networks.

**Figure 1:**
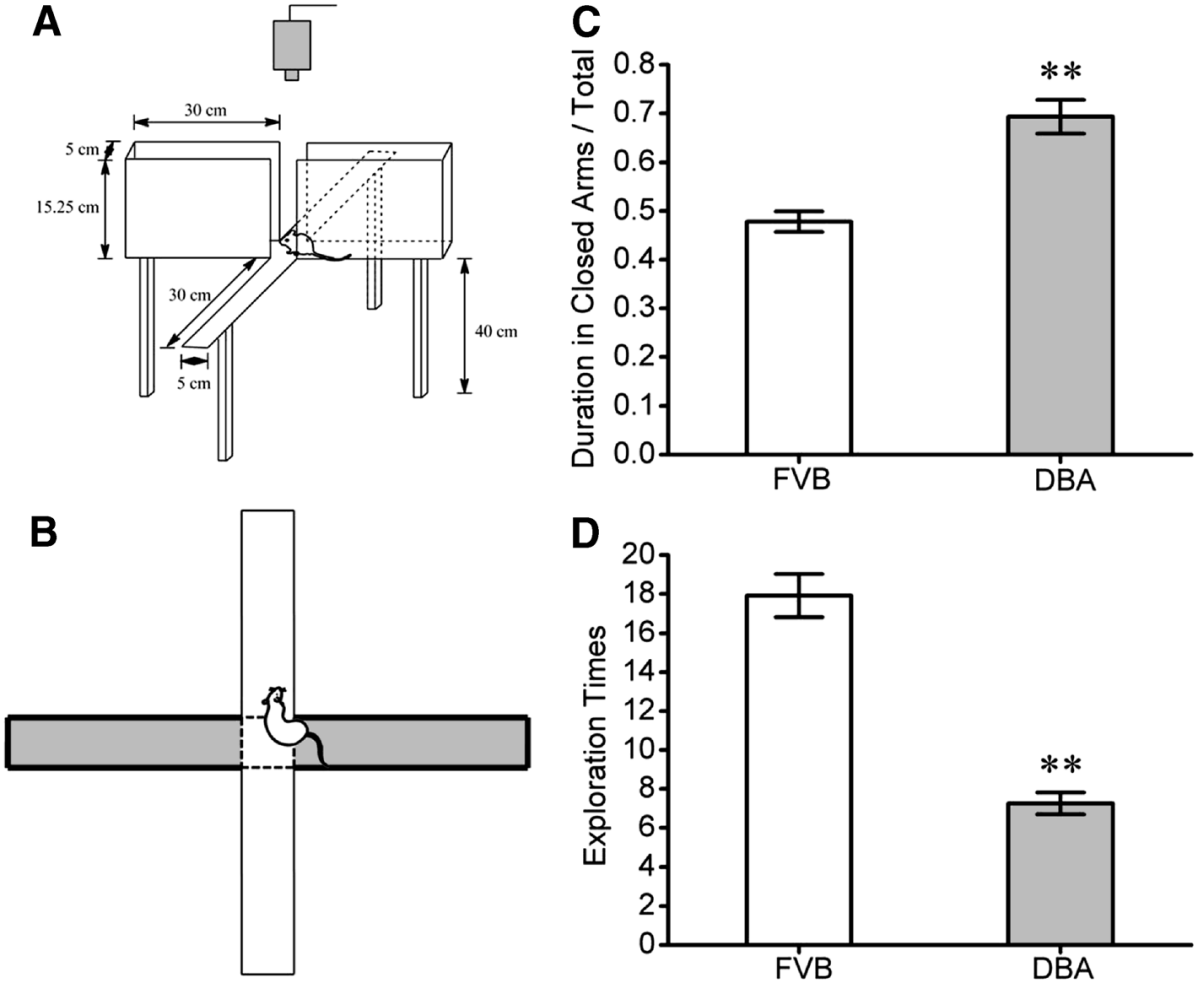
**DBA/2 mice show higher anxiety compared to FVB/N mice.** An elevated plus-maze was used to evaluate anxiety-like behavior in the strains of DBA/2 and FVB/N mice. **A**) The diagram shows the experimental methods to examine a mouse staying in the open arms vs. closed ones in an elevated plus-maze. **B**) shows the mouse position for counting exploration times. **C**) illustrates the duration for mice staying in the closed arms vs. total duration for each experiment in DBA/2 mice (gray bar) and FVB/N mice (white; p < 0.01, n = 12). **D**) shows exploration times towards the open arms for DBA/2 mice (gray bar) vs. FVB/N ones (white; p < 0.01, n = 12). The duration for staying in the closed arms is longer and the exploration times are lower significantly in DBA/2 mice than FVB/N mice.

Before used these mice, we first examined their anxiety-like behaviors with an elevated plus-maze [41,42]; and Figure 1A). High anxiety-like behavior is described as the mice spending more time in the closed arms and having less exploration times toward the open arms. In these measurements from DBA/2 and FVB/N mice, the duration of staying in the closed arms/total is longer in DBA/2 mice (gray bar) than FVB/N mice (white; p < 0.01, n = 12; Figure 1C). The exploration times toward the open arms are lower in DBA/2 mice (gray bar) than FVB/N ones (white; p < 0.01, n = 12; Figure 1D). The level of anxiety-like behaviors is higher in DBA/2 mice than FVB/N mice. This conclusion is consistent with a report about anxiety-like behaviors in different strains of mice [29]. 

### The activity asynchrony of network neurons in the amygdala of DBA/2 anxiety-like mice

The abnormality of amygdala is presumably one of major mechanisms associated with anxiety [1,15,22,38-40]. However, the pathological dynamics of its neuronal networks has not been characterized [35], which we examined in basolateral amygdala of DBA/2 high anxiety mice by two-photon cellular imaging and electrophysiology. Control experiments were conducted in FVB/N low anxiety mice. The temporal and spatial patterns in the activity of amygdala network neurons were evaluated by detecting the changes of intracellular Ca^2+^, since neuronal spikes raise its levels [43,44]. Oregon-green BAPTA-AM was loaded into the cells of brain slices including amygdala from DBA/2 and FVB/N mice to monitor intracellular Ca^2+^ levels. Sulforhodanmine-101 (SR-101) was used to label the astrocytes [45]. Fluorescents in amygdala were excited and detected by a two-photon laser scanning microscopy.

**Figure 2:**
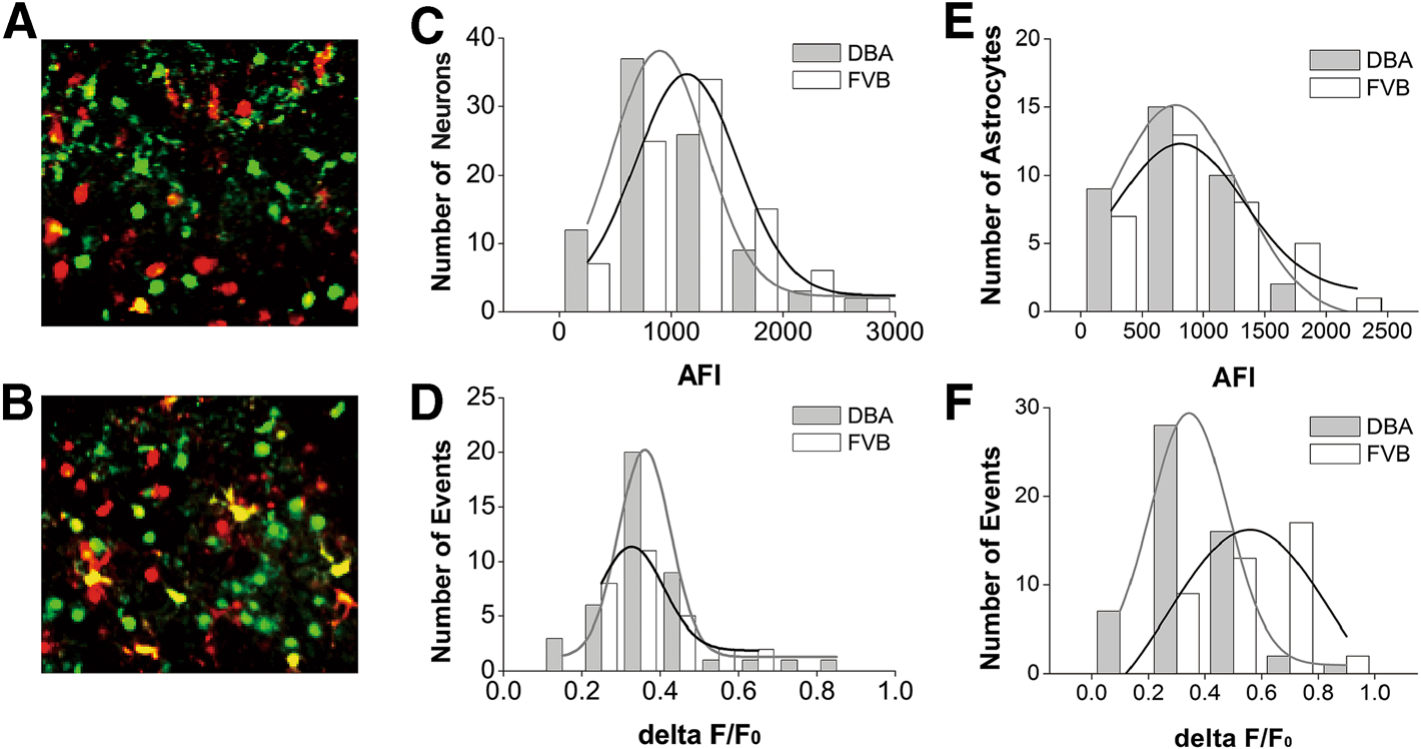
**The activity strength of amygdala neurons is not different in DBA/2 and FVB/N mice.** Oregon -green BAPTA-AM was loaded into the cells in brain slices including amygdala to monitor Ca^2+^ levels in neurons and astrocytes. Sulforhodanmine-101 was used to label astrocytes. Fluorescents in amygdala areas were excited and detected by two-photon laser scanning microscopy. **A**) shows a photo of Ca^2+^ imaging from the neurons (green) and astrocytes (red and yellow) in DBA/2 mice. **B**) shows Ca^2+^ imaging from the neurons (green) and astrocytes (red/yellow) in FVB/N mice. **C**) shows the number of neurons vs. their absolute fluorescence intensity (AFI) in DBA/2 mice (gray bars/fitting curve; n = 12 for mice and n = 27 for slices) and FVB/N mice (white bars/black curve; n = 11 for mice and n = 25 for slices). **D**) shows the number of spontaneous events from neurons versus their relative fluorescence intensity (ΔF/F_0_) in DBA/2 (gray bars/fitting curve) and FVB/N mice (white bars/black curve). **E**) shows the number of astrocytes vs. their absolute fluorescence intensity (AFI) in DBA/2 mice (gray bars/fitting curve; n = 12 for mice and n = 27 for slices) and FVB/N mice (white bars/black curve; n = 11 for mice and n = 25 for slices). **F**) shows the number of spontaneous events from astrocytes versus their relative fluorescence intensity (ΔF/F_0_) in DBA/2 (gray bars/fitting curve) and FVB/N mice (white bars/black curve).

**Figure 3:**
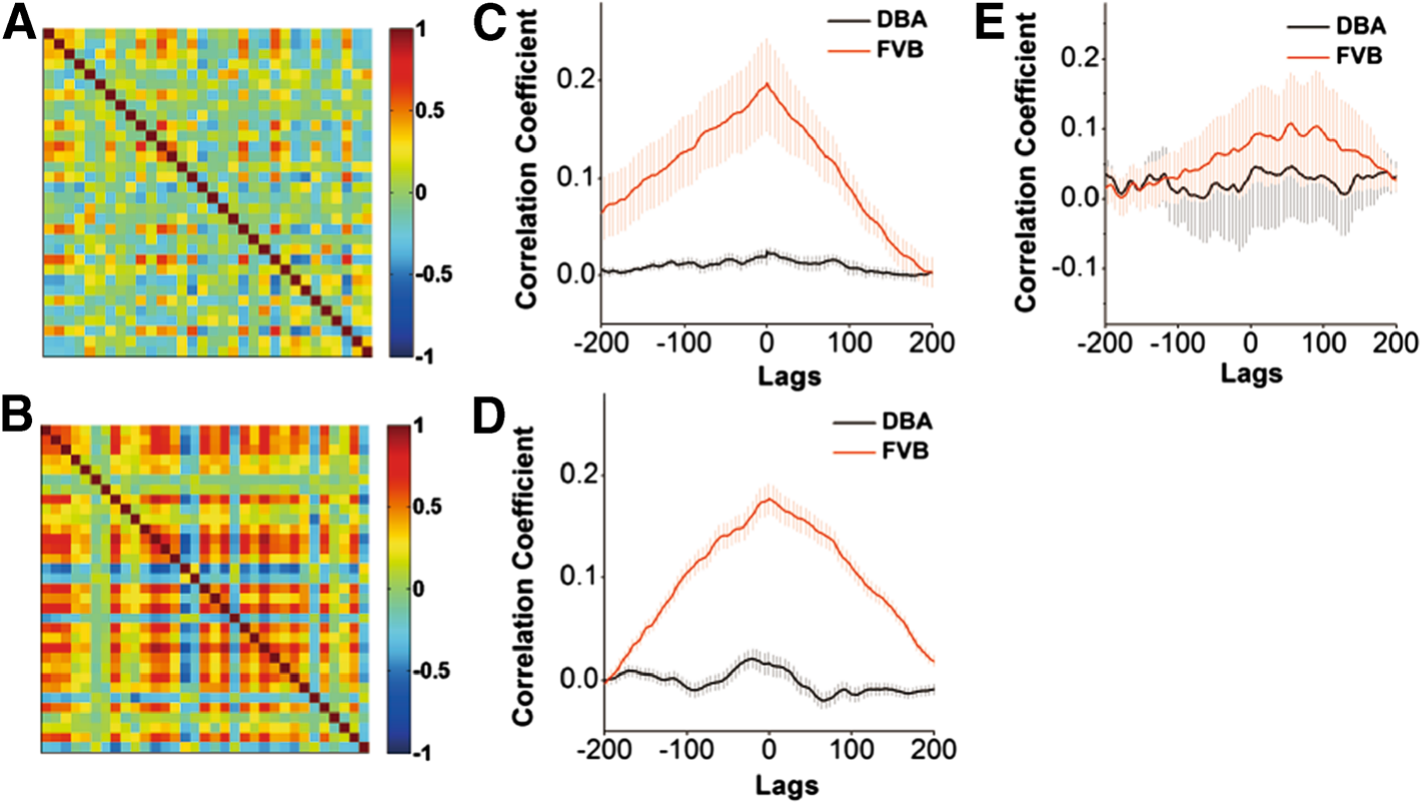
**The activities of amygdala neurons are less synchronous in DBA/2 mice than in FVB/N mice.** Two-photon cellular imaging was conducted under the frame scanning. **A**) The picture of chip patterns shows the cross-correlations in the timing phase of activity between two neighboring neurons in DBA/2 mice. Colors from red to blue indicate their cross-correlations from high (synchronous activity) to low. **B**) shows the cross-correlations between neighboring neurons in FVB/N mice. **C**) shows a comparison in the cross-correlations averaged from all of the visible amygdala neurons in a FVB/N mouse (red line) and in a DBA/2 mouse (black), in which experiments were done in a single day (p < 0.01). **D**) illustrates a comparison in the cross-correlations averaged from all of FVB/N mice (red line; n = 11) and DBA/2 ones (black; p < 0.01, n = 12). **E**) shows a comparison in cross-correlations averaged from all of the astrocytes in FVB/N mice (red line) and DBA/2 ones (black; p = 0.56).

Figures 2, 3 illustrate spatial and temporal patterns in the activity of amygdala network neurons from DBA/2 high anxiety and FVB/N low anxiety mice. Two-photon Ca^2+^ images in the neurons (green) and astrocytes (red/yellow) in amygdala slices from these mice are showed in Figure 2A-B, respectively. We analyzed their basal and spontaneous signals to present the activity strength of amygdala neurons, Figure 2C shows the number of cells versus their absolute fluorescence intensity (AFI) in DBA/2 (gray bars/fitting curve) and FVB/N mice (white/black), in which average values are 1059 ± 581 for DBA/2 mice (n = 12 for mice and n = 27 for slices) and 1307 ± 676 for FVB/N mice (n = 11 for mice and n = 25 for slices). Figure 2D shows the number of spontaneous events versus their relative fluorescence intensity (ΔF/F_0_) in DBA/2 (gray bars/fitting curve) and FVB/N mice (white/black). The averaged values are 0.376 ± 0.14 for DBA/2 mice and 0.378 ± 0.11 for FVB/N mice (p = 0.96, n = 42). There is no difference in the activity strength of amygdala neurons between DBA/2 high anxiety and FVB/N low anxiety mice. Similarly, the activity strength of astrocytes in amygdala between DBA/2 and FVB/N mice is not difference (Figure 2E-F). We subsequently analyzed the temporal activity properties of nerve cells in amygdala.

The temporal activity of amygdala neurons is evaluated by cross-correlations in the time phase of activities between neighboring neurons [46-48]; Methods). Chip patterns in Figure 3A-B show the cross-correlations of cell temporal activity from two strains of mice. The colors from red to blue denote their correlation coefficients from high (synchronous activity) to low (asynchronous). Amygdala neurons in DBA/2 high anxiety mice (3A) show lower cross-correlations than those in FVB/N low anxiety mice (3B). Figure 3C demonstrates correlation coefficients averaged from amygdala neurons in a FVB/N mouse (red line) and a DBA/2 (black, p < 0.01). Figure 3D illustrates the correlation coefficients averaged from all FVB/N mice (red line, n = 11) and DBA/2 mice (black; p < 0.01, n = 12). It is noteworthy that cross-correlations from all astrocytes in FVB/N mice (red line) and DBA/2 ones (black) are not statistically different (p = 0.56, Figure 3E). These data from analyzing cross-correlation indicate that the activities of amygdala neurons are less synchronous in DBA/2 high anxiety mice than FVB/N low anxiety mice.

**Figure 4:**
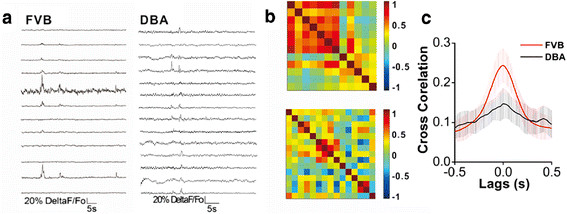
**The activities of amygdala neurons are less synchronous in DBA/2 mice than in FVB/N mice.** Two-photon cellular imaging was conducted under the line scanning. **A**) shows the comparisons in the events of Ca^2+^ signals linearly scanned in FVB/N mice (left panel) and DBA/2 mice (right). **B**) shows the cross-correlations in the timing phase of activity between two neighboring neurons in FVB/N mice (top panel) and DBA/2 mice (bottom). Colors from red to blue indicate their cross-correlations from high (synchronous activity) to low. **C**) shows the comparisons in the cross-correlations averaged from visible amygdala neurons in all of FVB/N mice (red line, n = 6 for mice and n = 11 for slices) and DBA/2 ones (black, n = 5 for mice and n = 12 for slices; p < 0.05).

We also conducted two-photon cellular imaging under the line scanning to examine the temporal activity of amygdala neurons in DBA/2 and FVB/N mice, in which the scanning rate reached above 50 Hz (Methods). Figure 4A shows the comparisons in the events of Ca^2+^ signals in FVB/N (left panel) and DBA/2 mice (right). The chip patterns in Figure 4B show cross-correlations in the timing phase of activity between two neighboring neurons in FVB/N (top panel) and DBA/2 mice (bottom). Colors from red to blue indicate their cross-correlations from high (synchronous activity) to low. Figure 4C compares the cross-correlations averaged from amygdala neurons in FVB/N mice (red line, n = 6 for mice and n = 11 for slices) and DBA/2 ones (black, n = 5 for mice and n = 12 for slices; p < 0.05). These data are consistent with those under the frame scanning, i.e., the activities among amygdala neurons are less synchronous in DBA/2 high anxiety mice than FVB/N low anxiety ones.

### Diversified neuronal excitability and weak GABAergic synapses in amygdala of DBA/2 anxiety-like mice

The activity asynchrony of amygdala neurons in DBA/2 anxiety-like mice may be caused by their functional diversity and GABAergic synapse weakness, since the activities of cerebral neurons are presumably coordinated by inhibitory interneurons [49-55]. We examined neuronal intrinsic properties and GABAergic synapses in the amygdala.

**Figure 5:**
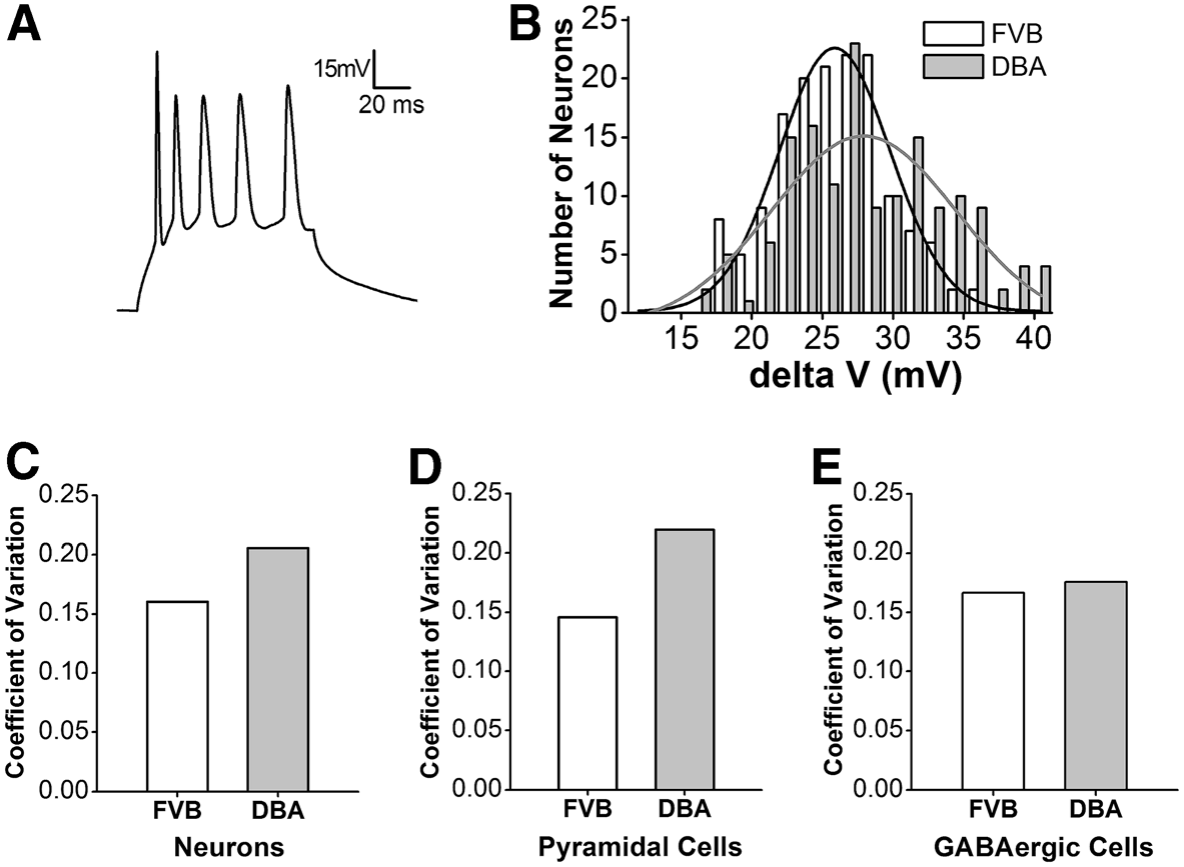
**Amygdala neurons in DBA/2 mice show more diversified excitability.** Neuronal excitability was assessed by a gap (ΔV) between threshold potential (Vts) and resting membrane potential (Vr), in which the membrane potentials were measured by whole-cell current-clamp recording in brain slices. **A**) shows the measurement and assessment of neuronal excitability from the recorded sequential spikes in an amygdala neuron. **B**) The histogram shows the number of neurons versus ΔV, i.e., the distributions of amygdala neuron excitability, from FVB/N mice (white bars) and DBA/2 mice (gray bars). The fitting curves in bell-shape are from FVB/N mice (black line) and DBA/2 mice (gray line). The wide curve in DBA/2 mice shows a diversified excitability. **C**) shows the coefficients of variance (standard deviation over mean) for ΔV of all amygdala neurons in FVB/N (white bar) and DBA/2 (gray bar). **D**) shows the coefficients of variance for ΔV of all amygdala non-fast spiking neurons in FVB/N (white bar) and DBA/2 (gray bar). **E**) shows the coefficients of variance for ΔV of amygdala fast spiking neurons in FVB/N (white bar) and DBA/2 (gray bar).

The sensitivity of amygdala neurons to inputs (i.e., threshold potentials) was used to merit their functional diversity [56,57]. The histogram in Figure 5B shows the number of neurons vs. threshold potential (ΔV), i.e., the distributions of amygdala neuron excitability, from FVB/N mice (white bars/black line for fitting curve, n = 153) and DBA/2 mice (grays, n = 153). A wide fitting-curve in DBA/2 mice indicates the diversity of neuronal excitability. Figure 5C presents the coefficient of variance (CV, standard deviation/mean) for ΔV from all amygdala neurons in FVB/N (white bar) and DBA/2 (gray) mice. Figure 5D illustrates CV for ΔV from amygdala non-fast spiking neurons in FVB/N (white bar, n = 80) and DBA/2 mice (gray, n = 80). Figure 5F illustrates CV for ΔV from amygdala fast-spiking neurons in FVB/N (white bar, n = 73) and DBA/2 mice (gray, n = 73). The excitability of amygdala neurons is diversified in DBA/2 high anxiety mice, leading to their asynchronous activities, especially for non-fast spiking neurons. 

**Figure 6:**
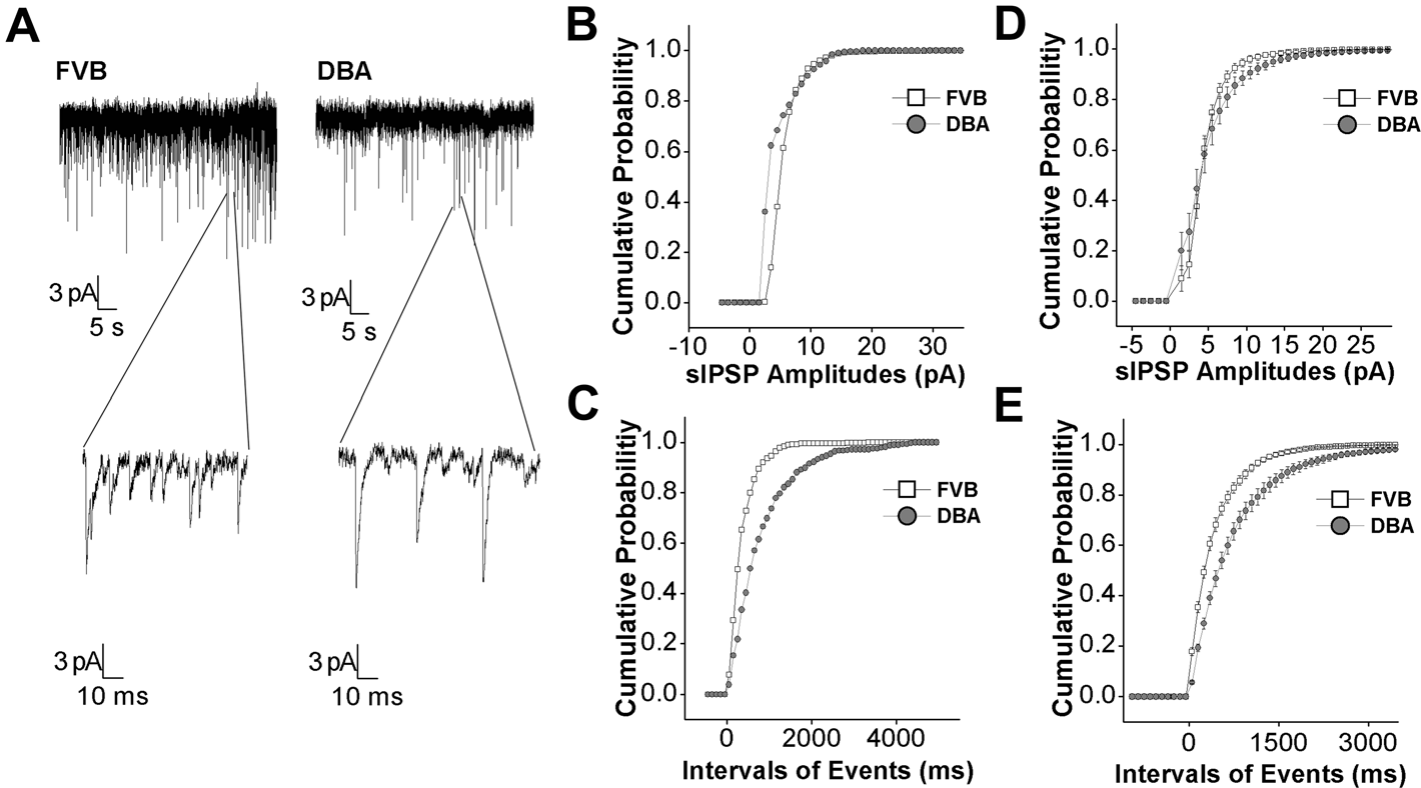
**GABAergic synaptic transmission is low in the amygdala neurons of DBA mice.** Spontaneous GABAergic inhibitory postsynaptic currents (IPSC) were recorded by whole-cell clamp at the amygdala neurons of brain slices from DBA/2 anxiety mice and control FVB/N mice. **A**) Left panels show sIPSCs recorded from FVB/N mice in top panel and sIPSCs with an extended time scale (bottom). Right panels show sIPSCs recorded from DBA/2 mice. **B**) shows cumulative probability vs. sIPSCs amplitudes from FVB/N mice (open symbols) and DBA/2 mice (filled ones) in an experiment of panel A. **C**) illustrates cumulative probability vs. intervals of events (sIPSCs) from FVB/N mice (open symbols) and DBA/2 mice (filled ones) in an experiment of panel A. **D**) shows cumulative probability vs. sIPSCs amplitudes in FVB/N mice (open symbols) and DBA/2 mice (filled) from all of the experiments (n = 12, p < 0.01). **F**) shows cumulative probability vs. intervals of events in FVB/N mice (open symbols) and DBA/2 mice (filled) averaged from all of the experiments (n = 11).

We also examined whether the weakness of GABAergic synaptic transmission was associated with the activity asynchrony of amygdala neurons in DBA/2 anxiety-like mice. Spontaneous inhibitory postsynaptic currents (IPSC) were recorded by whole- cell voltage-clamp at amygdala neurons of brain slices from DBA/2 and FVB/N mice. sIPSC events in Figure 6A were recorded in amygdala neurons from FVB/N mice (left panel) and DBA/2 mice (right). Cumulative probability versus sIPSC amplitudes and inter-event intervals analyzed from Figure 6A experiments in FVB/N (open symbols) and DBA/2 mice (filled) are presented in Figure 6B-C, respectively. Figure 6D illustrates cumulative probability versus sIPSC amplitudes averaged from all of the experiments in FVB/N (open symbols; n = 11) and DBA/2 mice (filled; n = 11). Figure 6E shows cumulative probability vs. inter-event intervals in FVB/N (open symbols) and DBA/2 (filled, n = 11, p < 0.01). Therefore, a weakness of GABAergic synaptic transmission at amygdala neurons is associated with DBA/2 anxiety-like mice.

The results above indicate that the activity asynchrony of neuronal activity, the diversity of their excitability and the weakness of GABAergic synapses in the amygdala are associated with anxiety pathophysiology. We subsequently seek the approach to improve anxiety-like behaviors and cellular pathophysiology. Temporal activities of cortical neurons are hypothetically regulated by metabotropic glutamate receptors (mGluR, [58]. We examined the influences of mGluR activation on neuronal network activity, GABAergic synaptic transmission and anxiety-like behaviors.

### mGluR activation improves anxiety, network asynchrony and GABA synaptic transmission

**Figure 7:**
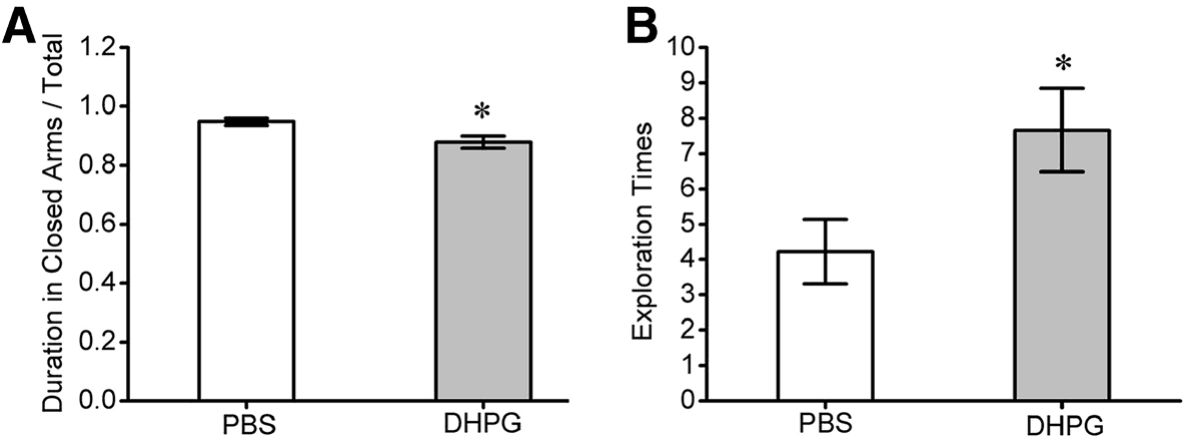
**The agonist of metabotropic glutamate receptors, 3,5-dihydroxyphenylglycine (3,5-DHPG), improves anxiety-like behavior in DBA mice.** An elevated plus-maze was used to evaluate anxiety-like behavior in two groups of DBA/2 mice that were given 3,5-DHPG (0.183 mg/kg) and PBS saline. **A**) shows the duration of staying in the closed arms vs. total duration for each experiment for DHPG-given mice (gray bar) versus PBS-given mice (white bar; p < 0.05, n = 9). **B**) shows exploration times toward the open arms in DHPG-given mice (gray bar) vs. PBS-given mice (white bar; p < 0.05, n = 9).

The effects of mGluR on anxiety and its neural pathophysiology were studied by using mGluR_1,5_ agonist, 3,5-dihydroxyphenylglycine (3,5-DHPG). DBA/2 mice were randomly divided to two groups that were given the intraperitoneal injections of 3,5-DHPG (0.183 mg/kg, one time per day) and PBS, respectively. Five days after this treatment, their anxiety-like behaviors were evaluated on an elevated plus-maze. The duration of staying in the closed arms is shorter in DHPG-given mice (gray bar) than PBS-given mice (white; p < 0.05, n = 9; Figure 7A). Exploration times toward the open arms are higher in DHPG-given mice (gray bar) vs. PBS-given mice (white; p < 0.05, n = 9; Figure 7B). The activation of mGluR_1,5_ improves anxiety-like behaviors in DBA/2 mice.

**Figure 8:**
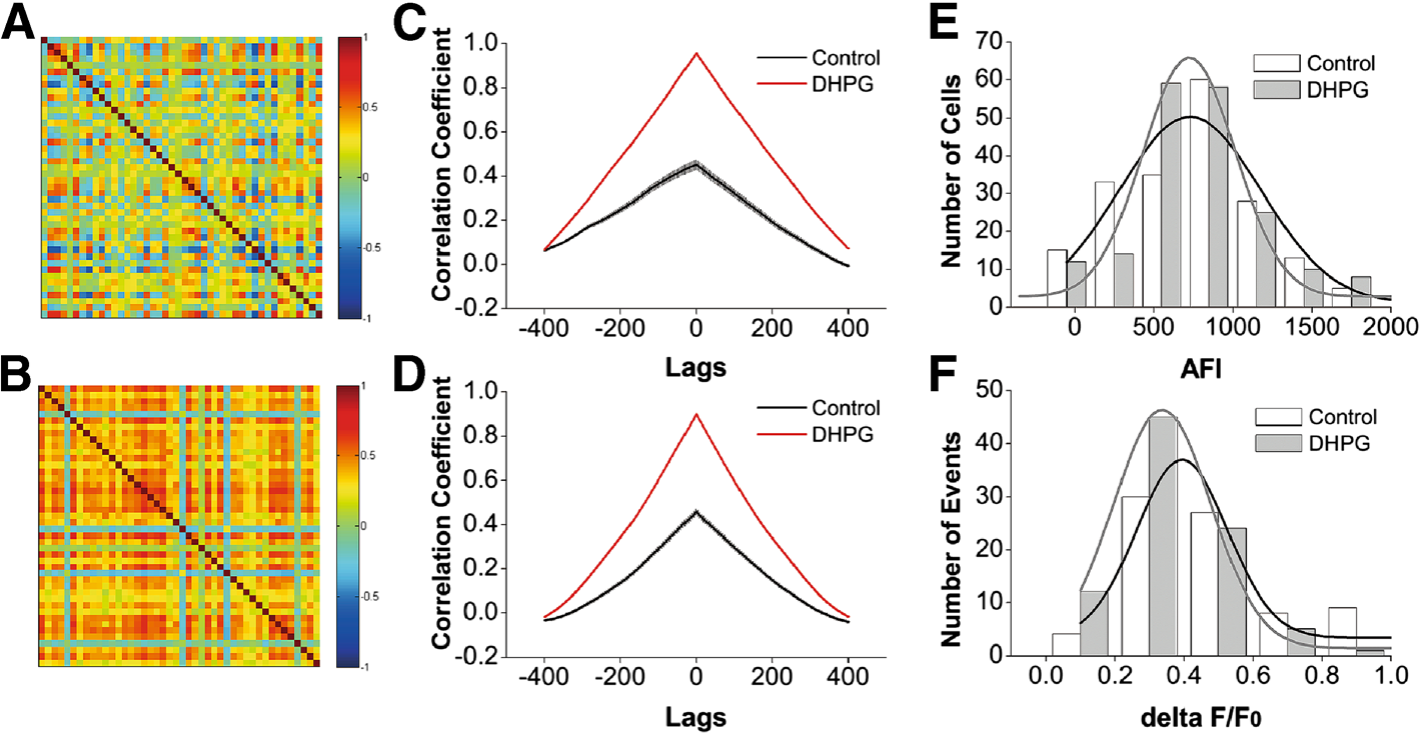
**The effect of 3,5-DHPG on the synchronous activity of amygdala neurons in DBA/2 mice.** Oregon-green BAPTA-AM was loaded into the cells in brain slices including amygdala to monitor Ca^2+^ levels in neurons. OGB-1 in amygdala were excited and detected by a two-photon laser scanning microscopy before and after DHPG (10 μM) was washed onto the slices. **A** ~ **B**) The pictures of chip patterns show the cross-correlations in the timing phase of activity between neighboring neurons under the conditions of control (A) and 3,5-DHPG wash-on (B). Color red presents a high cross-correlation (synchronous activity). **C**) shows a comparison in correlation coefficients averaged from all of the visible amygdala neurons before (black symbols/line) and after (red) washing 3,5-DHPG to a slice from a DBA/2 mouse (p < 0.01). **D**) illustrates correlation coefficients averaged from all of experimental DBA/2 mice (n = 7) before (black symbols and line) and after (red) washing-on DHPG (p < 0.01). **E**) shows the comparison in the number of cells vs. their absolute fluorescence intensity (AFI) before (gray bars/fitting curve) and after applying DHPG (white bars/black curve) in DBA/2 mice. **F**) shows a comparison in the number of spontaneous events versus their relative fluorescence intensity (ΔF/F) before (gray bars/fitting curve) and after applying DHPG (white bars/black curve) in DBA/2 mice.

If the asynchronous activity of network neurons is involved in anxiety-like behavior, we expect to see that mGluR activation synchronizes the activities of neurons in DBA/2 anxiety-like mice. We tested this assumption in the network neurons of amygdala slices, in which the temporal and spatial activity patterns of amygdala neurons were examined by two-photon cellular imaging (Method). Fluorescents in amygdala areas were excited and detected by a two-photon laser scanning microscopy before and after 3,5-DHPG (10 μM) was washed into the slices from DBA/2 mice. Chip patterns (cross-correlations, Figure 8A-B) show the time phase of activity between neighboring amygdala neurons from a DBA/2 mouse before and after using 3,5-DHPG. Colors from blue to red indicate their cross-correlations from low (asynchronous activity) to high (synchronous one). Figure 8C shows the correlation coefficients averaged from visible amygdala neurons in a slice from DBA/2 mouse under the conditions of control (black symbols/line) and DHPG (red, p < 0.01). Figure 8D illustrates the correlation coefficients averaged from all experimental DBA/2 mice (n = 6 for mice and n = 12 for slices) before (black symbols/line) and after (red) applying 3,5-DHPG (p < 0.01). Thus, the activation of mGluR_1,5_ improves the asynchronous activity of amygdala network neurons in DBA/2 anxiety-like mice.

In addition, the influences of mGluR_1,5_ activation on the activity strength of amygdala neurons in DAB/2 anxiety-like mice are showed in Figure 8E-F. Figure 8E illustrates the comparison in the number of cells vs. their fluorescence intensity in DBA/2 mice before (gray bars/fitting curve) and after (white bars/black curve) using 3,5-DHPG, in which the average values are 773 ± 488 for control and 825 ± 512 for DHPG (p = 0.3, n = 193). Figure 8F shows the number of spontaneous events versus their relative fluorescence intensity in DBA/2 mice before (gray bars/fitting curve) and after (white bars/black curve) 3,5-DHPG, in which the average values are 0.512 ± 0.26 for control and 0.38 ± 0.23 for 3,5-DHPG. Thus, mGluR_1,5_ activation does not affect the activity strength of amygdala network neurons in DBA/2 anxiety-like mice.

**Figure 9:**
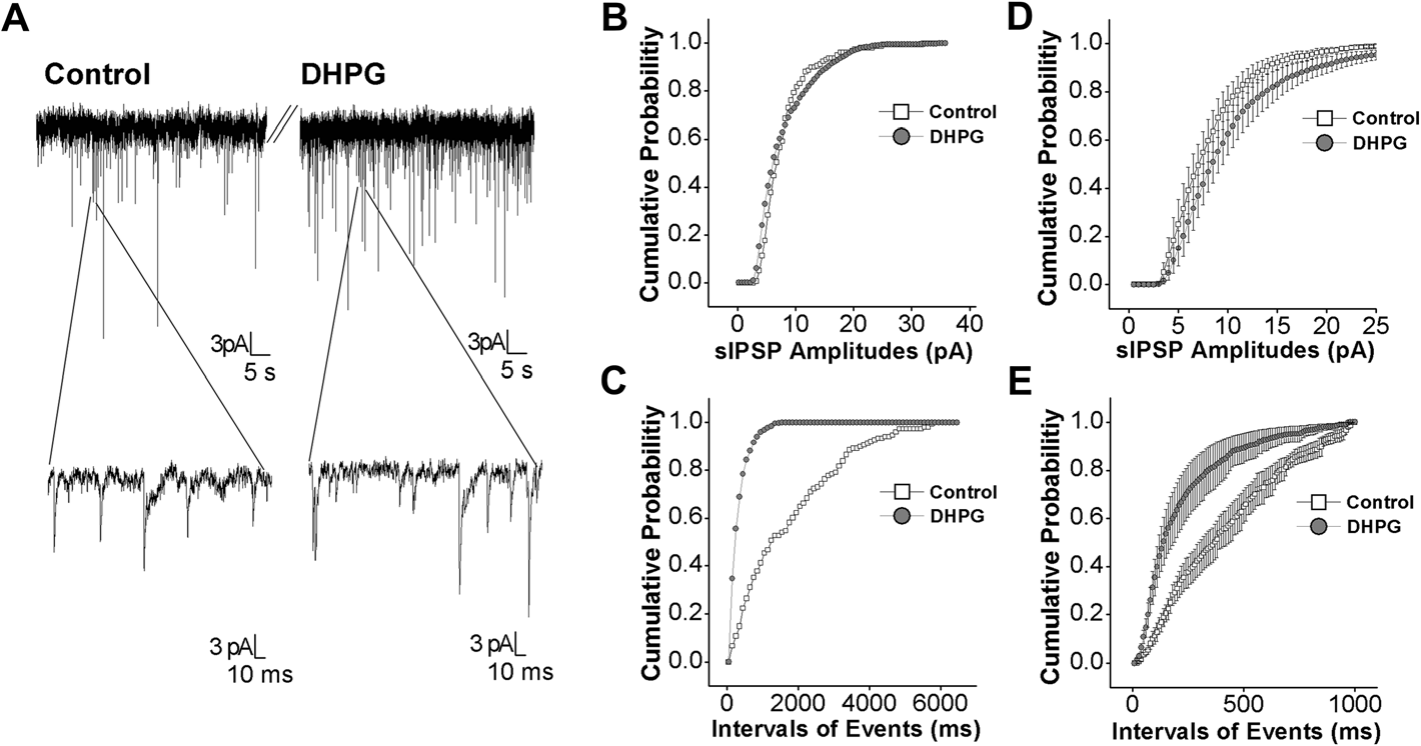
**3,5-DHPG enhances GABAergic synaptic transmission in the amygdala neurons of DBA mice.** Spontaneous GABAergic inhibitory postsynaptic currents (IPSC) were recorded by whole-cell clamp at amygdala neurons in brain slices before and after DHPG (10 μM) was washed onto the slices. **A**) shows GABAergic sIPSCs under the conditions of control (left panels) and DHPG application (right panels). Bottom panels show sIPSCs in the extended time scales. **B**) shows cumulative probability vs. sIPSCs amplitudes before (opened symbols) and after using DHPG (filled ones) from an experiment in panel A. **C**) shows the cumulative probability vs. inter-event intervals for sIPSCs before (opened symbols) and after applying DHPG (filled ones) from an experiment in panel A. **D**) shows the cumulative probability vs. sIPSCs amplitudes before (opened symbols) and after applying DHPG (filled ones) averaged from all of the experiments (n = 8). **E**) shows the cumulative probability vs. inter-event intervals for sIPSCs before (opened symbols) and after using DHPG (filled ones) from all of the experiments (n = 8, p < 0.01).

If the synchronous activity of network neurons is coordinated by GABAergic neurons, we should see that mGluR activation strengthens GABA synaptic transmission in amygdala neurons of DBA/2 anxiety-like mice. Spontaneous inhibitory postsynaptic currents (sIPSC) were recorded by whole-cell voltage-clamp at amygdala neurons in brain slices before and after 3,5-DHPG (10 μM) was washed on. Figure 9A illustrates sIPSCs recorded before (left panels) and after applying 3,5-DHPG (rights). Cumulative probability versus sIPSC amplitudes and inter-event intervals from 9A experiment before (open symbols) and after using 3,5-DHPG (filled) are showed in Figure 9B-C. Figure 9D shows cumulative probability vs. sIPSC amplitudes averaged from all of the experiments (n = 7) before (open symbols) and after 3,5-DHPG (filled ones). Figure 9E illustrates cumulative probability vs. inter-event intervals before (open symbols) and after 3,5-DHPG (filled, n = 7, p < 0.01). Thus, mGluR_1,5_ activation enhances GABAergic synaptic transmission in the amygdala neurons of DBA/2 anxiety-like mice.

## Discussion

We studied cellular mechanisms underlying anxiety-like behavior in DBA/2 mice by using two-photon cellular imaging, electrophysiology and behavioral analysis. The temporal asynchrony of neuronal activity and the weakness of GABAergic synaptic transmission in amygdala are associated with anxiety-like behavior (Figures 2, 3, 4, 5 and 6). mGluR_1,5_ activation improves anxiety-like behavior, synchronizes neuronal activities and enhances GABAergic synaptic transmission in the amygdala (Figures 7, 8 and 9). These results indicate mGluR_1,5_ agonists may be potentially useful as anxiety therapies. Our studies bring insights into the pathological mechanisms between genetic deficits and anxiety-like behavior, and the potential medications for anxiety disorders.

Previous studies imply that the abnormality of certain genes is associated with anxiety disorders, such as CRHR1, FKBP5, CREB, Egr-1, Glo1, Gsr, AC8, CaMKIV, dystrophin, HTTLPR and COMT Met158 [25-34,59]. The amygdala dysfunction is presumably one of anxiety pathogeneses [1,10,14-16,20,22,24]. The cellular processes linking genes’ defects and functional impairment in amygdala remain unclear. We used DBA/2 mice that are genetic variances (glyoxalase-1/glutathione reductase-1 genes) and anxiety-like phenotype [29] to study the cellular pathophysiology of anxiety. Our studies will be extended to address the relationships between other genes and anxiety-related cellular pathophysiology.

Neural hyperactivity in amygdala may be associated with anxiety disorders [5,6,60,61], and its networks are hypothetically abnormal [1,15,22,35]. Compared to these studies by brain imaging [5,6,60,61], two-photon imaging in our studies has cellular resolution, which enables the temporal and spatial activity natures of individual cells be analyzed. Here, we reveal that the amygdala neurons in DBA/2 anxiety-like mice appear a temporal asynchrony in their activities (Figures 3 and 4). As GABAergic inhibitory system coordinates the activities of network neurons [49,53-55], the dysfunction of GABAergic synapses in amygdala (Figure 6) leads to the asynchrony of neuronal network. Our studies by imaging cell dynamics provide a direct evidence for this hypothesis, and address its mechanisms.

Previous studies indicate that dysfunctional interactions in ligands and receptors, e.g., serotonin, norepinephrine, GABA, glutamate and hormones, are associated with anxiety [62-65]. The reagents strengthening the efficacy of their interactions were applied for the psychotropic medication of anxiety disorders, such as selective serotonin reuptake inhibitors, tricyclic antidepressants, monoamine oxidase inhibitors and benzodiazepine [66]. Despite notable advances, numerous patients suffering from anxiety disorders fail to adequately respond to these pharmacologic reagents. In addition, the objects to strengthen ligand-receptor interactions may be realized by mGluR-mediated activations of cellular signals. Figures 7, 8 and 9 indicate that mGluR_1,5_ activation improves anxiety-like behavior, synchronizes neuronal activity and enhances GABA synaptic transmission in the amygdala of DBA/2 mice, indicating a potential use of mGluR agonists for the therapy of anxiety disorders. This supports an early-stage effort to understand the role of mGluR reagents in anxiety disorders [67-69].

**Figure 10:**
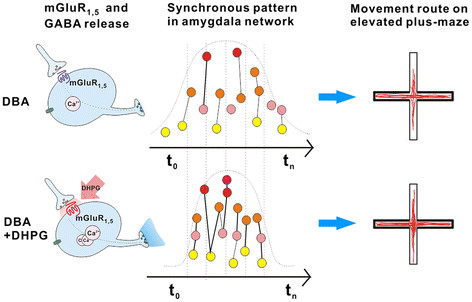
**A diagram illustrates a potential mechanism underlying the enhancement of GABAergic synaptic transmission, the improvement of synchronous activities of amygdala neurons as well as the attenuation of anxiety-like behavior in DBA/2 mice by DHPG, an agonist of metabotropic glutamate receptor.** Left column shows the influence of mGluR activation on GABAergic synaptic transmission (bottom panel), compared to control (top). DHPG elevates intracellular Ca^2+^ and enhances GABA release by activating mGluR_1,5_. Middle column illustrates that activity synchrony among amygdala neurons is improved when GABAergic synapses are enhanced by DHPG. Compared with top panel, temporal activity in amygdala neurons (bottom) in the presence of DHPG tends toward synchrony in time scale. Right column illustrates that anxiety-like movement routes on an elevated plus-maze in DBA/2 mice (top panel) are improved by DHPG (bottom).

In terms of role of mGluR in regulating the synchrony of network neurons and the transmission of GABAergic synapses, we draw a testable hypothesis in Figure 10. Metabotropic glutamate receptors as G-protein coupled receptor activate in intracellular Ca^2+^-signaling cascades, which may enhance the release of GABA. The strengthening of GABAergic synaptic transmission in turn coordinates the temporal activities of amygdala neurons.

It is noteworthy that the values measured in behavioral tasks from DBA mice are variable in Figure 1 vs. Figure 7. As all of the treatments to mice were identical, only difference between these two groups was intraperitoneal injections. In addition, the injections of 3,5-DHPG and PBS saline increased the duration in closed arms/total (Figure 7), compared with no injection (Figure 1). Therefore, our explanation for such discrepancies would be that the injections may influence anxiety-like behaviors. This explanation is supported by a reference in behavioral study [70].

In conclusion, mGluR_1,5_ activation improves anxiety-like behavior by up-regulating GABAergic synapse-mediated synchrony of amygdala network neurons (Figure 10). Our findings are adding the knowledge for the pathological mechanisms and medications of anxiety disorders.

## Methods and materials

### The selection of mice with anxiety-like behavior

According to the study in mouse behavior [29], we selected two strains of mice DBA/2 and FVB/N, which demonstrate high anxiety and low anxiety, respectively (also see Figure 1). Mice in postnatal day (PND) 20 ~ 30 were used to conduct the experiments of behavioral tasks as well as of two-photon cellular imaging and electrophysiology in amygdala of brain slices. The elevated plus-maze was used to evaluate anxiety-like behaviors. Patch-clamp was used to record the transmission of GABAergic synapses and the intrinsic property of amygdala neurons. A two-photon laser scanning microscopy was applied to image the activities of neural networks in the amygdala. The entire procedures are approved by the Institutional Animal Care Unit Committee in the Administration Office of Laboratory Animals Beijing China (B10831).

### Behavioral study

Anxiety-like behaviors in DBA/2 and FVB/N mice were evaluated by an elevated plus-maze (EPM), which is described as a validated and classic method to assess the level of anxiety in the rodents [41,42]. The EPM consists of two open arms (30 × 5 cm) opposite to two closed arms (30 × 5 × 15.25 cm). The arms extended from a central platform (5 × 5 cm). The EPM was located 40 cm above the floor (Figure 1A). FBV/n and DBA/2 mice were housed in plastic cages with food/water availability *ad libitum* and a schedule of alternative light and dark (12 hours for each condition), in which the light was on 17:00*.* All experiments were performed between 8:00 to 14:00. Mice were at the age about one month when the tests were conducted.

Mice naturally avoid the open field. On the other hand, they intend to explore a new environment for food. In this regard, the measurement for mice avoiding the open field was the duration when mice stayed in the closed arms, i.e., the duration in the closed arms vs. total experimental time, whereas the measurement for mice exploring the new environment was exploration times toward the open arms (Figure 1B). Therefore, exploration times and the duration in the closed arms were used to evaluate the level of anxiety, which were recorded by an automatic video-tracking system for 5 min. Mice were placed at the open field of an elevated plus-maze with facing to a closed arm at the beginning of experiments. High anxiety-like behaviors are described as mice spending more time in the closed arms as well as having lower exploration times toward the open arms. Behavioral data were presented as means ± SE and statistically analyzed by one-way ANOVA (Origin Lab).

### Electrophysiology study

Brain slices in coronal section including cortex, amygdala, hippocampus and thalamus (350 μm) were prepared from DBA/2 and FVB/N mice. These mice were anesthetized by injecting chloral hydrate (300 mg/kg) and decapitated by a guillotine. The slices were sectioned by a Vibratome in the modified and oxygenized (95% O_2_ and 5% CO_2_) artificial cerebrospinal fluid (mM: 124 NaCl, 3 KCl, 1.2 NaH_2_PO_4_, 26 NaHCO_3_, 0.5 CaCl_2_, 5 MgSO_4_, 10 dextrose and 5 HEPES; pH 7.35) at 4 °C, and then were held in the normal oxygenated ACSF (mM: 124 NaCl, 3 KCl, 1.2 NaH_2_PO_4_, 26 NaHCO_3_, 2.4 CaCl_2_, 1.3 MgSO_4_, 10 dextrose and 5 HEPES; pH 7.35) 25 °C for 1–2 hours. A slice was transferred into a submersion chamber (Warner RC-26 G) that was perfused with normal ACSF at 31°C for electrophysiological experiments [71-73].

Neurons in basolateral amygdala were recorded by whole-cell clamp under a visualized condition (DIC/FN-E600, Nikon, Japan). Spontaneous inhibitory postsynaptic currents (sIPSC) from GABAergic synapses were recorded by voltage-clamp model (MultiClamp 700B and pClamp 10, Axon Instrument, Foster CA, USA) on amygdala neurons. Standard pipette solution contained (mM) 135 K-gluconate, 20 KCl, 4 NaCl, 10 HEPES, 0.5 EGTA, 4 Mg-ATP, and 0.5 Tris–GTP. The osmolarity of pipette solutions was 295–310 mOsmol, and the resistance of filled pipettes was 5 ~ 7 MΩ. Based on Nernst equation, the concentration of chloride ions in this pipette solution makes reversal potential approximately −43 mV, which is consistent with values in our measurements. When we held the membrane potential at −70 mV, GABAergic sIPSCs were inward (down-fluctuation). Series and input resistances for all of the neurons were monitored by injecting hyperpolarization pulses (5 mV/50 ms) throughout each experiment, and calculated by voltage pulses versus instantaneous and steady-state currents. 6-Cyano-7-nitroquinoxaline −2,3-(1 *H*,4 *H*)-dione (10 μM) and D-amino-5-phosphonovanolenic acid (40 μM) were added into ACSF to block ionotropic receptor-channels in the glutamatergic synapses [74,75]. These procedures isolate GABAergic IPSCs out. At the end of experiments, bicuculline (10 μM) was washed onto the slices to test whether synaptic responses were purely mediated by GABA_A_R. Bicuculline did block synaptic currents recorded in our experiments.

Action potentials were recorded by MultiClamp-700B amplifier and inputted into pClamp10 with 100 Hz sampling rate (Axon Instrument Inc., Foster CA, USA). Transient capacitance was compensated, and output bandwidth filter was 3 kHz. Standard pipette solution contained (mM) 150 K-gluconate, 5 NaCl, 0.4 EGTA, 4 Mg-ATP, 4 Na-phosphocreatine, 0.5 Tris-GTP and 10 HEPES (pH 7.4 adjusted by 2 M KOH). Pipette solution osmolarity was 295–305 mOsmol, and pipette resistance was 6 ~ 8 MΩ. The threshold potentials (Vts) of sequential spikes, which were the voltages of firing sequential spikes, were measured [76-80].

Data were analyzed if the recorded neurons had resting membrane potentials negatively more than 60 mV. The criteria for the acceptation of each experiment also included less than 5% changes in the resting membrane potential, spike magnitudes, and input/seal resistance. The values of sIPSCs and Vts are presented as mean ± SE. The comparisons of the data from behavior tasks, electrophysiology and cellular imaging between groups are statistically done by one-way ANOVA.

### Cellular imaging

Ca^2+^ indicative dye was AM esters of its dye (Oregongreen BAPTA-1-AM) and astrocyte indicator was sulforhodanmine 101-AM (SR101; [45], in which AM element facilitated the dyes to be loaded into brain cells in slices. OGB1-AM was dissolved in DMSO and 20% Pluronic F-127 (2 g Pluronic F-127 in 10 ml DMSO) to have their stock solutions at 1 mM, and then were diluted in the oxygenated ACSF to yield its final concentration at 10 μM. SR-101 was dissolved in distilled water at 1 mM for stock solution and then dissolved in ACSF to 1 μM for the final concentration. These dyes in such solutions were loaded into the neurons and astrocytes in amygdala slices were based on a modified method [81]. A slice was placed in an incubation chamber (1 cm in diameter) containing 2 ml of the loading solution at 35 °C for 45 min, and the loading solution was then washed out with the oxygenated ACSF. A slice was transferred to a submersion chamber (Warner RC-26 G) and perfused by the oxygenated ACSF at 2 ml/min for cellular imaging experiments.

The images of OGB-1 for Ca^2+^ in amygdala neurons and astrocytes and SR-101 for astrocytes were taken by using a two-photon laser scanning microscope (Olympus FV-1000, Olympus, Tokyo Japan). The 2PLSM was equipped by a two-photon laser-beam generator (Mai Tai, Physical Spectrum, USA) and a scanning system mounted onto an upright microscope (Olympus BX61WI) with water immersion objectives (40X, 0.8NA). A laser beam (810 nm) was given to excite OGB-1-AM and SR-101. The emission wave spectra were 523 nm for Ca^2+^-binding OGB-1 and 603 nm for SR-101, respectively. Average power delivered to the brain slices was <10 mW. The parameters set for the laser beam and photomultiplier tube were locked for two groups of slices throughout the experiments in order to have consistent condition in the comparisons of the results between DBA/2 and FVB/N mice. Images were viewed and analyzed with Fluoviewer. Data are presented as the changes in fluorescence intensity [82].

Both frame scanning and line scanning were applied for the imaging. The pixels crossed with a manually drawn line in the interested cells were scanned under the line scanning, in which the scanning rate reached as high as 50–200 Hz. In line scan, these areas were scanned 512 × 512 pixels. In the frame scan, 400 × 400 μm areas in amygdala slices were scanned by 320 × 320 pixels, and the rates were 5–10 frames/s. It is noteworthy that in consistence with the duration of a single spike and sequential spikes, Ca^2+^ signals can be classified into two phases, fast and slow phases. Based on the temporal resolution in our experiments, Ca^2+^ signals were activated neuronal sequential spikes. All of the frames in an independent and complete scanning acquired by a software fluoview were exported as a single movie file which was then analyzed with ImageJ software (National Institute of Health) or custom-made codes in Matlab (MathWorks).

All fluorescence signals were acquired by using Fluoviewer-10 software (Olympus Inc. Japan) and analyzed from the cell bodies in amygdala. Signals are presented as relative fluorescence change [ΔF/F = (F-F_basal_)/F_basal_] after subtracting background noise from unstained blood vessels. F is the fluorescence intensity at any time point, and F_basal_ the baseline fluorescence averaged across appointed time course or the whole movie for each cell.

Cross-correlations among fluorescent signals have been used to present the temporal correlations in the activity of network nerve cells [46-48]. Here, we analyzed the pairswise cross-correlation of Ca^2+^ signals in amygdala neurons and astrocytes between each of pairs to indicate their temporal dynamics. The correlation coefficients normalized to the autocorrelation at zero lag were calculated. Considering two signals x(t) and y(t) of a real variable t, we defined the correlation coefficients *r* at delay *d*

(1) $$r=\frac{\sum \left[\left(x\left(t\right)-mx\right)\times \left(y\left(t-d\right)-my\right)\right]}{\sqrt{\sum_{t}{\left(x\;\left(t\right)-mx\right)}^{2}}\times \sqrt{\sum_{t}{\left(y\;\left(t-d\right)-my\right)}^{2}}}$$

*mx* and *my* are the means of the corresponding series. Based on these calculations, the correlation matrices were plotted using MATLAB 7.0. All of the data are presented as mean ± SD. Student’s t tests (two-tailed, paired, or unpaired assuming unequal variances) were done in R software package, version 2.10.1 (http://www.r-project.org/), to make the statistical evaluation. A *p* value ≤ 0.05 was defined as a statistical significance.

## Competing interests

The authors declare that they have no competing interests.

## Authors’ contributions

FYZ, BL and ZFL conduct experiments and data analyses, and JHW contributes to experimental designs and paper writing. All authors read and approved the final manuscript.
